# Genome sequencing reveals a splice donor site mutation in the *SNX14* gene associated with a novel cerebellar cortical degeneration in the Hungarian Vizsla dog breed

**DOI:** 10.1186/s12863-016-0433-y

**Published:** 2016-08-26

**Authors:** Joe Fenn, Mike Boursnell, Rebekkah J. Hitti, Christopher A. Jenkins, Rebecca L. Terry, Simon L. Priestnall, Patrick J. Kenny, Cathryn S. Mellersh, Oliver P. Forman

**Affiliations:** 1 Department of Clinical Science and Services, Royal Veterinary College, Hawkshead Lane, North Mymms, Hertfordshire, AL9 7TA UK; 2 Department of Pathology and Pathogen Biology, Royal Veterinary College, Hawkshead Lane, North Mymms, Hertfordshire, AL9 7TA UK; 3 Kennel Club Genetics Centre, Animal Health Trust, Kentford, Newmarket, Suffolk, CB8 7UU UK

**Keywords:** Cerebellar cortical degeneration, Cerebellar abiotrophy, Hungarian Vizsla dog, Genome sequencing

## Abstract

**Background:**

Cerebellar cortical degeneration (CCD) is an increasingly recognised neurodegenerative disease process affecting many dog breeds. Typical presentation consists of a progressive cerebellar ataxia, with a variable age at onset and rate of progression between different breeds. Cerebellar histopathological findings typically consist of primary Purkinje neuronal degeneration and loss, with variable secondary depletion of the granular and molecular cell layers. Causative genes have been identified associated with CCD in several breeds, allowing screening for selective breeding to reduce the prevalence of these conditions. There have been no previous reports of CCD in Hungarian Vizslas.

**Results:**

Two full-sibling Hungarian Vizsla puppies from a litter of nine presented with a history of progressive ataxia, starting around three months of age. Clinical signs included marked hypermetric and dysmetric ataxia, truncal sway, intention tremors and absent menace responses, with positional horizontal nystagmus in one dog. Routine diagnostic investigations were unremarkable, and magnetic resonance imaging performed in one dog revealed mild craniodorsal cerebellar sulci widening, supportive of cerebellar atrophy. Owners of both dogs elected for euthanasia shortly after the onset of signs. Histopathological examination revealed primary Purkinje neuron loss consistent with CCD. Whole genome sequencing was used to successfully identify a disease-associated splice donor site variant in the sorting nexin 14 gene (*SNX14*) as a strong causative candidate. An altered *SNX14* splicing pattern for a CCD case was demonstrated by RNA analysis, and no SNX14 protein could be detected in CCD case cerebellum by western blotting. SNX14 is involved in maintaining normal neuronal excitability and synaptic transmission, and a mutation has recently been found to cause autosomal recessive cerebellar ataxia and intellectual disability syndrome in humans. Genetic screening of 133 unaffected Hungarian Vizslas revealed the presence of three heterozygotes, supporting the presence of carriers in the wider population.

**Conclusions:**

This is the first report of CCD in Hungarian Vizsla dogs and identifies a highly associated splice donor site mutation in *SNX14*, with an autosomal recessive mode of inheritance suspected.

**Electronic supplementary material:**

The online version of this article (doi:10.1186/s12863-016-0433-y) contains supplementary material, which is available to authorized users.

## Background

Cerebellar cortical degeneration (CCD) has been reported in several different dog breeds [1–15], and is also commonly referred to as cerebellar abiotrophy. CCD typically causes slowly progressive clinical signs related to diffuse cerebellar dysfunction, including a dysmetric and hypermetric cerebellar ataxia, intention tremors, menace response deficits and signs of central vestibular dysfunction, including nystagmus and loss of balance [12, 16]. Age of onset of clinical signs and the rate of progression vary between breeds, ranging from neonatal, as reported in Beagles and Rhodesian Ridgebacks [3, 13], and early onset from weeks to months of age in the majority of breeds [2, 6, 8, 9, 14, 16], up to adult-onset at months to years of age with a slower progression, seen in Old English Sheepdogs, Gordon Setters and Scottish Terriers [12, 15, 17].

Histopathological examination most often reveals a primary diffuse loss of Purkinje neurons in the cerebellar cortex, in some cases with milder secondary neuronal depletion of the granular and molecular cell layers [7, 11, 13, 14, 16]. Less commonly there have also been reports of primary granular cell loss with sparing of the Purkinje neuron layer in some dog breeds, including the Border Collie and Bavarian Mountain Dog [6, 8], as well as an immune-mediated granuloprival degeneration in the Coton de Tulear [18]. Neurodegenerative lesions in some dog breeds with CCD have been reported to also affect areas of the central nervous system outside the cerebellum, as seen in the Kerry Blue Terrier and Chinese-Crested Dog [19, 20].

An autosomal recessive mode of inheritance for CCD is suspected in most breeds, and putative causative genetic mutations have been identified for CCD in several of these [13–15, 21]. A mutation in the *GRM1* gene was identified as the cause of Bandera’s neonatal cerebellar ataxia in the Coton de Tulear, which represents a functional disturbance with minimal histopathological evidence of CCD [21]. A recent study used genome-wide mRNA sequencing to identify a causative mutation in the β-III spectrin cytoskeletal protein gene, *SPTBN2*, in Beagle dogs with neonatal CCD [13]. Genome-wide association studies also recently identified a mutation in the *SEL1L* gene, encoding a component of the endoplasmic reticulum-associated protein degradation machinery, in Finnish Hounds with CCD [14], as well as a mutation associated with adult-onset CCD in Gordon Setters and Old English Sheepdogs in the autophagy gene, *RAB24* [15]. This variation in clinical presentation, histopathology and molecular findings suggests that CCD represents a heterogeneous disease process with varied underlying genetic aetiologies in different dog breeds.

Following the diagnosis of CCD in two Hungarian Vizsla (HV) littermates, the aim of this study was to describe the clinical and histopathological features of CCD previously unreported in this breed and to interrogate the genome of an affected dog for potential causal mutations.

## Methods

### Clinical investigation

Two full-sibling HVs (Case 1 and 2) from a litter of nine were presented separately to the Royal Veterinary College (RVC), University of London for further investigation of progressive ataxia and tremors. Clinical examinations and diagnostic tests were performed at the RVC, with videos obtained of both examinations. Whole blood was taken ante-mortem from both dogs and stored for future molecular investigations. The dogs were both euthanased for welfare reasons at their owners’ request, and submitted for post mortem examination. Histopathological examination of the brain tissue was performed on both dogs. Samples of cerebellar tissue from the male sibling (Case 1) were obtained and stored in RNAlater medium to allow subsequent RNA extraction. Relevant clinical history was obtained where possible from the breeder regarding the unaffected littermates, dam and sire.

Full post-mortem examination was performed in both dogs, with samples of brain tissue preserved in 10 % neutral buffered formalin followed by embedding in paraffin wax. 4 μm thick sections were cut on a microtome (Leica Jung RM2035) and stained with haematoxylin and eosin. Slides were evaluated by a board-certified veterinary pathologist (SLP), using light microscopy.

### Molecular investigation

DNA was extracted from whole blood using the Nucleon BACC2 DNA extraction kit (GE Healthcare Life Sciences). RNA was extracted from canine cerebellum using the Qiagen RNeasy midi kit (Qiagen), with on column DNase treatment.

Whole genome resequencing of a single HV with CCD (Case 2) was outsourced to the Wellcome Trust Centre for Human Genetics, University of Oxford. Paired end sequencing with a read length of 100 bp read generated a dataset of approximately 75 Gb, resulting in ~30× coverage of the genome. Reads were aligned to the CanFam3.1 canine genome build using Burrows-Wheeler Aligner (BWA) [22] and variants called using Genome Analysis Toolkit (GATK) [23]. Variants were filtered based on consequence predictions created using SNPeff [24], and by comparison with variant calls generated for 13 unrelated genome sequences of other dog breeds.

A library for RNAseq was prepared using total RNA extracted from Case 1. Messenger RNA was isolated using Sera-Mag oligo(dT) magnetic beads, and libraries prepared using the NEBNext® mRNA Sample Prep Master Mix Set 1. The final RNA-seq library was quantified by qPCR using the Kapa library quantification kit (Kapa Biosystems). Paired-end sequencing of 80 bp reads was carried out on an Illumina MiSeq, producing an ~4Gb dataset. Reads were aligned to the canine reference genome (CanFam3.1) using BWA and further processed using GATK. Aligned reads were viewed using The Integrative Genomics Viewer (IGV).

Genotyping of the CCD associated variant was performed using an allelic discrimination approach (Taqman), using an ABI StepOne real-time thermal cycler. Primer sequences were as follows: SNX14_F ATTACTGTATATTGTGATCTCAAAGAAATGCTAATCT; SNX14_R, AGCAAAAACGAGCAAAACAGACTTT; SNX14_V, VIC-ACATTCCAGGTATAATATT-NFQ; SNX14_M FAM-ACATTCCAGATATAATATT-NFQ.

Reverse transcription PCR was carried out in two stages. Reverse transcription was performed using the Qiagen Quantitect cDNA synthesis kit. PCR was performed in 12 μl reactions consisting of 0.2 mM dNTPs (NEB), 1× PCR buffer (Qiagen), 0.5 μM of each primer (SNX14_x25F, CCGCTTGGTCTCACTCATAAC and SNX14_x29_R, CACAATGTCCAATAAAACATAAGTCA), 0.5 units HotStarTaq plus DNA polymerase (Qiagen), and template cDNA. Cycling parameters were 95 °C for 5 min, followed by 35 cycles of 95 °C for 30 s, 57 °C for 30 s and 72 °C for 60 s, and a final elongation stage of 72 °C for 10 min. Sanger sequencing was performed using BigDye v3.1 dye terminator chemistry.

For western blotting, cerebellum tissue from the affected HV case and 6 unaffected control dogs (one each of the following breeds: Golden Retriever, Crossbreed, Beagle, Parson Russell Terrier, Great Dane and Labrador Retriever cross) were first homogenized in lysis radioimmunoprecipitation assay buffer (RIPA, Sigma-Aldrich, R0278). 5 μl (46–56 μg) of the total protein lysate was separated by 8 % SDS-polyacrylamide gel electrophoresis. Proteins were then transferred onto nitrocellulose membrane, and immunoblotting was performed using rabbit polyclonal anti-SNX14 antibody (1:500, Atlas Antibodies, HPA017639) and swine polyclonal anti-rabbit antibody conjugated with horseradish peroxidase (HRP) (1:1000, Daco, P0217). The protein bands were detected using Novex ECL (Thermo Fisher Scientific) HRP chemiluminescent substrate reagent kit (WP20005) with high performance chemiluminescence film (Amersham Hyperfilm ECL). To control for protein loading the same protein lysates were used in the same quantities for a separate western blot using mouse monoclonal anti-beta Actin antibody (1:1000, Abcam, mAbcam 8226) and goat polyclonal anti-mouse antibody conjugated with HRP (1:1000, Daco, P0447).

## Results

### Clinical investigation

**Fig. 1:**
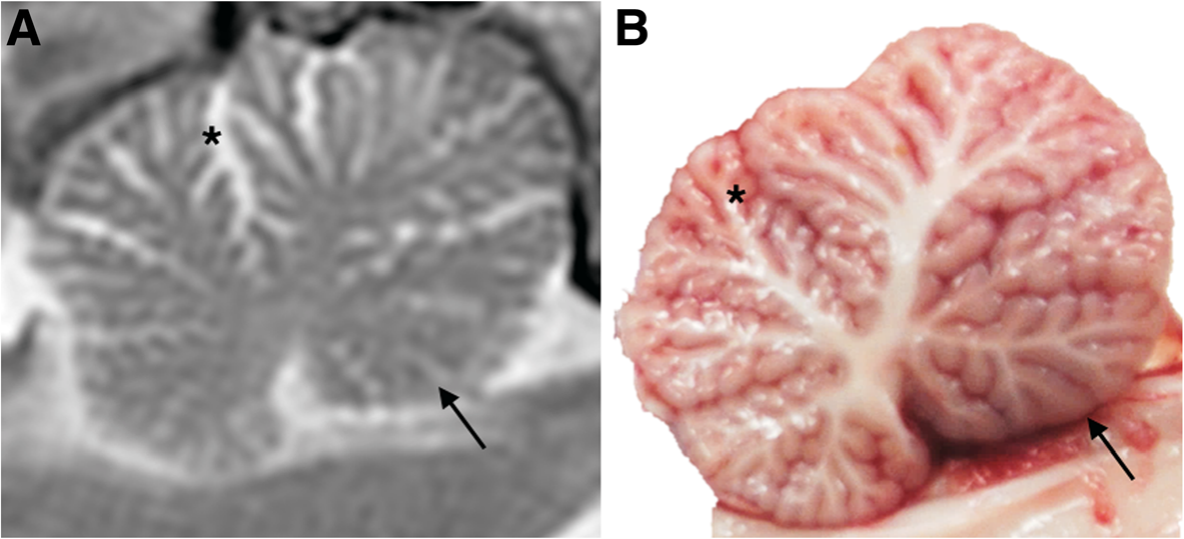
Cerebellar MRI and gross pathological specimen from an HV with CCD (Case 1). Midline sagittal T2-weighted MRI image (**a**), and fresh sagittal section (**b**), of the cerebellum of a 4-month old HV with CCD. Predominantly affecting the rostral and dorsal cerebellum, there is widening of the cerebellar sulci with increased cerebrospinal fluid (increased T2-weighted signal) between the folia and subjectively reduced cortical grey matter (*asterisks*). This is not evident in the caudoventral lobules, which appear grossly normal (*arrows*)

Cases 1 (male) and 2 (female) both presented aged four months old, having first demonstrated signs of ataxia between two and three months of age. Until this time they had developed the ability to walk at a similar rate to their littermates. By four months of age the ataxia had worsened and Case 1 had developed a marked head tremor, more obvious with intention. The dogs were otherwise bright and well, with normal behaviour reported by their owners and no other clinical signs reported. General physical examination was unremarkable on presentation to the RVC. On neurological examination of both dogs, mentation was alert and appropriate. They were both ambulatory with a markedly hypermetric and dysmetric generalised cerebellar ataxia, with an obvious intention tremor particularly when navigating their environment and excited, both of which were more exaggerated in Case 1. Paw positioning was intact, although hopping responses showed a delayed onset of limb protraction and exaggerated response in all limbs. Spinal reflexes were intact in all four limbs. Cranial nerve examination revealed absent menace responses bilaterally, with an intermittent positional horizontal nystagmus in Case 2 when her head was elevated. No hyperaesthesia was detected on spinal or cranial palpation. All other components of the neurological examination were within normal limits. These findings were consistent with a diffuse and symmetrical lesion affecting the cerebellum. Differential diagnoses included neurodegenerative disease, such as cerebellar cortical degeneration or lysosomal storage disorders, inflammatory/infectious disease, metabolic disease and neoplasia. Diagnostic investigations were performed for Case 1 but not Case 2, whose owners elected for euthanasia, with consent for post-mortem examination. Haematology, serum biochemistry and urinalysis in Case 1 revealed no significant abnormalities for a four month-old dog. Blood smear evaluation of leukocytes revealed no evidence of intracellular storage vacuoles. Cranial magnetic resonance imaging (MRI) revealed mildly increased width and depth of the craniodorsal cerebellar sulci, with the impression of a reduced volume of cerebellar grey matter tissue in this region (Fig. 1). The gross size of the cerebellum otherwise appeared largely normal, with a mid-sagittal cross-sectional brainstem to cerebellar area ratio of 81.1 % [25]. There were also mildly enlarged cerebral sulci and lateral ventricles, with the rest of the brain MRI otherwise unremarkable. Cerebrospinal fluid (CSF) sampled from the cerebellomedullary cistern was unremarkable, with low nucleated cellularity and protein. Based on the unremarkable blood tests, imaging findings and clinical history, a presumptive diagnosis of CCD was made. Following a deterioration in Case 1’s clinical signs in the following weeks, his owners also elected for euthanasia and consented to post-mortem examination. There were no similar clinical signs reported in the littermates or the parents of the affected HV siblings.

**Fig. 2:**
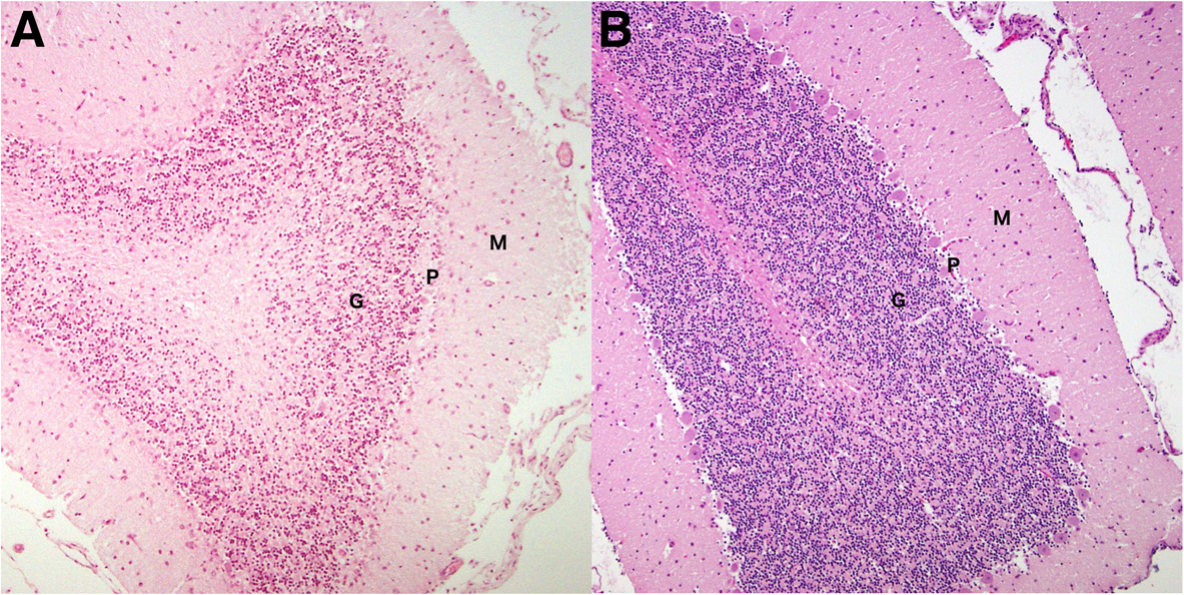
Histological findings in the cerebellar folia of an affected HV (Case 2, **a**) and a normal control dog (**b**). The affected HV (**a**) demonstrates marked loss of Purkinje neurons (P), with an associated secondary depletion and thinning of the granular cell layer (G). The unaffected control dog (**b**) cerebellar folium demonstrates the normal cellularity of the granular (G), Purkinje (P) and molecular (M) cell layers. (Haematoxylin and eosin stain, ×100 magnification)

**Fig. 3:**
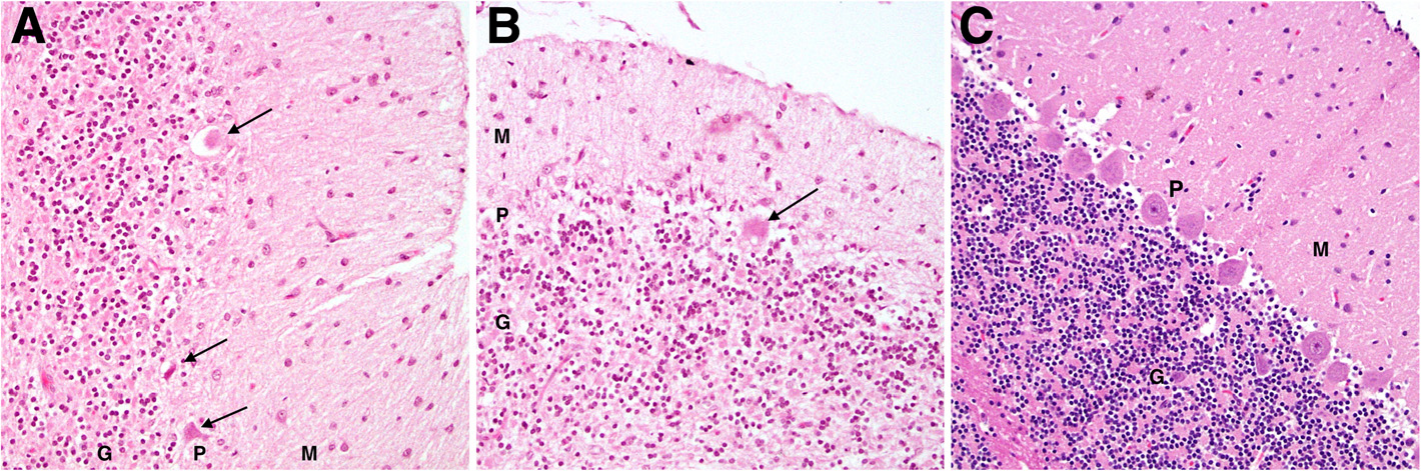
Cerebellar Purkinje neuron degeneration in an affected HV case (Case 2). Higher magnification of the cerebellar cortex in an affected HV (**a** and **b**) reveals shrunken, angular and eosinophilic (degenerate) Purkinje neurons (*arrows*). Reduced cellularity of the granular cell layer (G) and thinning and pallor of the molecular cell layer (M) are also evident, compared to the densely populated granular cell layer and normal molecular cell layer in the unaffected control dog (**c**). (Haematoxylin and eosin stain, ×200 magnification)

On gross examination, in both dogs the cerebellum appeared subjectively small relative to total brain size, with bilaterally symmetrical atrophy of the cerebellar hemispheres and a relative prominence of the cerebellar vermis. On sagittal section the cerebellar folia were reduced in thickness in both dogs (Fig. 1). The resulting diffuse widening of the cerebellar sulci was most prominent in the dorsal and rostral vermis, with relative sparing of the caudoventral lobules (Fig. 1). Microscopically, in both dogs there was multifocally extensive loss of cerebellar Purkinje neurons, which was particularly severe in Case 1 (Fig. 2). Some Purkinje neurons remained in Case 2 however they were frequently degenerate (Fig. 3). In both dogs there was a marked depletion of the granular cell layer, with a diffuse mild to moderate gliosis, and atrophy of the molecular layer (Figs. 2 and 3). There were rare degenerate neurons and spheroid formation in the cerebellar nuclei of Case 1. No evidence of inflammation or neoplasia was seen and the gross and histopathological examination of both dogs was otherwise unremarkable, with no histological evidence of intracellular material consistent with a lysosomal storage product.

### Genetic investigation

**Fig. 4:**
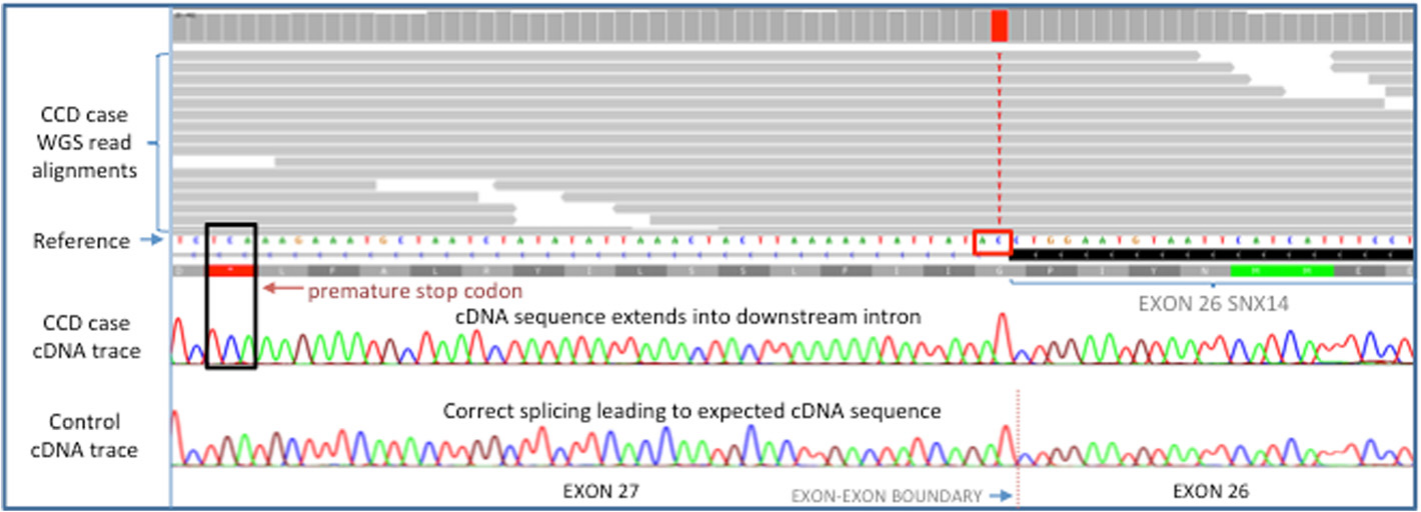
Splice donor mutation in CCD cases causing an altered splicing pattern. An exon 26 splice donor site mutation (GT > AT) causing exon 26 of *SNX14* to extent by 275 additional nucleotides to an alternative splice donor site. The extended exon has a sequence of 15 aberrant amino acids before a premature stop codon

The entire genome of a single HV with CCD (Case 2) was interrogated using a massively parallel sequencing approach. Paired end Illumina sequencing was performed to give a final average coverage of 30× on alignment to the canine genome sequence CanFam3.1. Variants identified in the HV were compared with variants identified for 13 additional canine breeds. Based on the hypothesis that the causal variant for CCD was rare and unique to the HV dog breed, variants were filtered to only include ones that were homozygous in the HV, and homozygous for the alternative allele in the 13 other breeds. Of the remaining variants 290 were predicted to either directly alter the coding sequence of the protein or disrupt the transcript sequence. A shortlist of 10 variants in potential candidate gene was generated by performing an automated search of the Online Mendelian Inheritance in Man database (OMIM) for the gene names implicated by the 290 variants and the key word cerebellar ataxia. The 10 shortlisted variants were individually considered by visually inspecting genome sequence read alignments, strength of the candidate gene, variant consequence (SIFT) and Case 1 genotype for each variant, established through an RNAseq dataset. All shortlisted variants, including an initially compelling variant in Ataxin1 (*ATXN1*), were excluded apart from an exon 26 splice donor variant (CanFam3.1, chr12:45,530,566, c.2653 + 1G > A) in the Sorting Nexin 14 (*SNX14*) gene; an excellent candidate causal variant (Fig. 4). A summary of the shortlisted variants is shown in the supporting information Additional file 1: Table S1. The potential causal variant was further interrogated by genotyping the second case and a set of 133 unaffected HV as controls. The other CCD case (Case 1) was also found to be homozygous for the disease-associated allele. Of the controls, three heterozygotes were identified with the remaining 130 HVs homozygous for the reference allele. Of the three heterozygotes, two were half-siblings and the other was unrelated at the parent level. Pedigree information for the CCD cases was not available, but no clinical signs were reported in either parent, supporting carrier status.

**Fig. 5:**
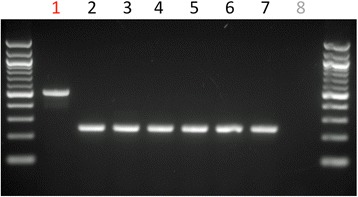
Altered splicing pattern in a CCD case. Polymerase chain reaction of cerebellar cDNA between exon 25 and 29 demonstrates a product size increase of 275 bp for the CCD case (1) in comparison to 6 control dogs (2–7) when visualised by agarose gel electrophoresis. No template control (8)

**Fig. 6:**
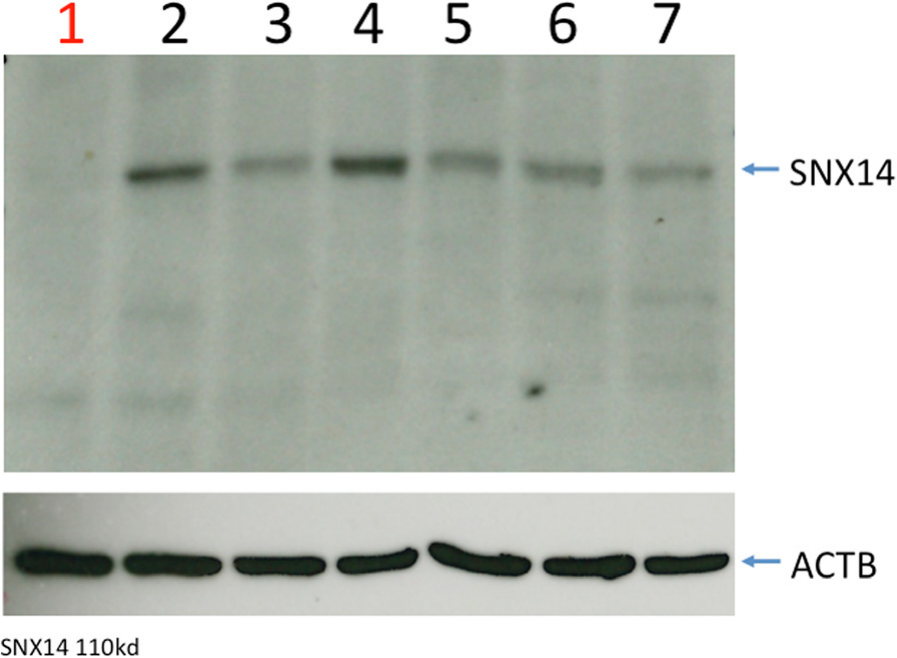
Western blot analysis of SNX14 protein. Western blot analysis of SNX14 failed to detect protein in cerebellar tissue of the CCD case (1). Presence of a band of expected size for SNX14 (110 kD) was detected for 6 canine control cerebellar samples (2–7). ACTB was used as a loading control

The effect of the variant on the *SNX14* transcript was investigated by RT-PCR across the exon 26–27 junction, using forward and reverse primers in exon 25 and 29 respectively and cerebellar RNA from one CCD case (Case 1) and the six control dogs. A larger than expected PCR product was seen for the CCD case (Fig. 5), with exon 26 in the case extending 275 bp to the position of an alternative splice donor site, resulting in an aberrant string of 15 amino acids and a premature stop codon (p.G885Dfs*16) (Fig. 4). Western blot analysis using an antibody targeting an amino acid sequence before the splice variant, failed to identify SNX14 protein (either full length or reduced) in cerebellum tissue of the CCD case (Fig. 6).

## Discussion

In this paper we present clinical, diagnostic, pathological and genetic findings associated with, to our knowledge, the first reported cases of CCD in HV dogs. We also report the use of genome sequencing to successfully identify a disease-associated splice donor site variant in *SNX14* as a strong candidate for CCD in the HV. An altered *SNX14* splicing pattern for a CCD case was demonstrated by RNA analysis and no SNX14 protein could be detected in CCD case cerebellum by western blotting.

The age of onset of clinical signs in the two reported HV dogs is consistent with previous reports of canine CCD in those breeds with a typically juvenile onset, with most of these cases having an onset at around 3–4 months of age, and a relatively rapid progression over several weeks to months [2, 6, 8, 9, 14, 16]. This is in contrast to breeds where a neonatal, rapidly progressive phenotype is seen, such as the Beagle and Rhodesian Ridgeback [3, 13], and those with a later onset and more slowly progressive condition typified by CCD in Gordon Setters and Old English Sheepdogs [15]. Similar to reports of canine CCD seen in other breeds [12, 16], the observed clinical signs in the HV were referable to diffuse cerebellar dysfunction, with both dogs displaying a marked hypermetric, dysmetric ataxia, with marked truncal sway and intention tremor, as well as bilateral menace deficits with normal vision. Although they remained ambulatory, both dogs demonstrated a progressive clinical course over a short period of around two months, to the point that their owners elected for euthanasia on humane grounds due to their poor quality of life. This history of progression to euthanasia is typical of CCD in most breeds, apart from those with an adult onset and slowly progressive CCD, whereby survival for years is possible [7, 12, 15].

Diagnostic investigations performed in Case 1 were consistent with CCD, with MRI revealing widening of sulci in the craniodorsal cerebellum suggesting atrophy of cerebellar grey matter in this area (Fig. 1), similar to previous MRI reports [5, 16, 25, 26]. The MRI scan performed in this HV with CCD revealed a mid-sagittal cross-sectional brainstem to cerebellar area ratio of 81.1 %, a value lower than a previously reported lower limit of 89 % for the diagnosis of cerebellar atrophy using MRI [25]. However, the 13 dogs used in that MRI study consisted mainly of breeds with an adult onset, slowly progressive form of CCD. The HV in the current study also underwent MRI early in the disease, and therefore given the variability in the time point in the disease course at which MRI is performed, comparison with dogs in the previous study is difficult. Given the uncertainty regarding the length of time required for cerebellar atrophy to be detectable on MRI in cases of CCD [25], it is possible that MRI was performed in Case 1 before sufficient atrophy to cause a reduction in MRI area ratio had developed.

Histopathological examinations in both dogs revealed diffuse Purkinje neuron loss, with secondary depletion of the granular cell layer, as well as reactive changes (gliosis) likely secondary to this degeneration of the Purkinje neuron layer. These changes fit well with previous reports of CCD or cerebellar abiotrophy, where primary Purkinje neuron loss and secondary granular cell degeneration are most commonly reported [7, 11, 13, 14, 16]. Similar to previous studies, the HVs also demonstrated a characteristic distribution of cerebellar folial atrophy, with the dorsal and rostral vermis predominantly affected whilst the caudoventral regions appeared spared [10, 12, 14, 17]. Purkinje neuron loss and associated gliosis were more severe in Case 1. In addition Case 1 also exhibited early neuronal degeneration within the cerebellar nuclei, which may be associated with the progression of clinical signs in this animal prior to euthanasia. These microscopic changes were sufficient to cause mild grossly observable symmetrical atrophy of the cerebellar hemispheres and thinning of the cerebellar folia, consistent with previous reports [1, 7, 16].

Mutations in *SNX14* have been reported to cause autosomal recessive cerebellar ataxia and intellectual disability syndrome in humans [27]. In the first report of an association between this syndrome and mutations in *SNX14*, three distinct *SNX14* mutations were identified which segregated in three unrelated consanguineous families, and included a nonsense mutation located in exon 26 of *SNX14*. Interestingly *SNX14* was investigated in the third family purely based on phenotypic similarities to the first two families. Full clinical signs included intellectual disability, cerebellar ataxia, early-onset cerebellar atrophy, sensorineural hearing loss, and the distinctive association of progressively coarsening facial features (broad face, fullness of the upper eyelid, broad nasal base and slight underdevelopment of the alae, broad and long philtrum, thick lower lip vermillion), relative macrocephaly, and the absence of seizures. Fifth finger brachycamptodactyly was also reported. There were no obvious distinctive facial phenotypic differences between CCD cases and unaffected HVs, and auditory function was normal on examination. However, electrodiagnostic tests of auditory function, such as brainstem auditory evoked potentials, could be performed in future studies to exclude a possible mild hearing loss. The intellectual disability seen in humans with *SNX14* mutations was also not identified in the HVs with CCD, although this is challenging to objectively evaluate in dogs and may have developed at a later age. Marked cerebellar ataxia and cerebellar atrophy with thinning of the cerebellar folia on gross pathology were seen in HV CCD cases, consistent with human cases.

SNX14 is a conserved 946 amino acid protein. In a knockdown mouse model, SNX14 has been shown to be involved in synaptic transmission and maintaining normal neuronal excitability [28]. Furthermore, using an in vivo *SNX14* translation blocking in a zebrafish model, loss of neuronal tissue and reduced cerebellar area was demonstrated, which could be rescued by co-injection with the human *SNX14* orthologue [29]. Cellular fractionation experiments have shown *SNX14* to be associated with the lysosomal fraction [29]. *SNX14* has also been shown to associate with the serotonin subtype 6 receptor, which has been implicated in cognition, anxiety and learning and memory disorders, with *SNX14* acting as a negative regulator [30].

Although not directly examined, there were no reported clinical signs in the remaining seven HV siblings or the parents, supporting an autosomal recessive mode of inheritance of the *SNX14* mutation in the HV, as reported in humans. Whilst we are not aware of any other cases of CCD yet identified in other HV families, the finding of three heterozygotes amongst the 133 unaffected HV samples confirms the presence of carriers in the wider HV population. As a result, genetic testing for the *SNX14* mutation could be beneficial both in supporting a presumptive antemortem diagnosis of CCD in an HV with consistent clinical signs, as well as allowing breeders to identify carriers.

## Conclusion

This paper represents the first confirmed report of cerebellar cortical degeneration in two young HV dogs and identifies a splice donor site mutation in *SNX14* as the likely cause, based on RNA analysis, protein analysis and studies in other species and in vivo models. The identification of this mutation will allow the development of a genetic test to help to identify affected and carrier dogs, to help to eliminate the disease from the breed.

## Additional file

Additional file 1:
**Table S1.** A summary of the 10 shortlisted variants in potential candidate genes. (XLSX 14 kb)

## Abbreviations

CCD: Cerebellar cortical degeneration
HV: Hungarian Vizsla
MRI: Magnetic resonance imaging
SNX14: Sorting nexin 14
